# Emergence of Azole-Resistant *Aspergillus fumigatus* Strains due to Agricultural Azole Use Creates an Increasing Threat to Human Health

**DOI:** 10.1371/journal.ppat.1003633

**Published:** 2013-10-24

**Authors:** Anuradha Chowdhary, Shallu Kathuria, Jianping Xu, Jacques F. Meis

**Affiliations:** 1 Department of Medical Mycology, Vallabhbhai Patel Chest Institute, University of Delhi, Delhi, India; 2 Department of Biology, McMaster University, Hamilton, Ontario, Canada; 3 Department of Medical Microbiology and Infectious Diseases, Canisius Wilhelmina Hospital, Nijmegen, The Netherlands; 4 Department of Medical Microbiology, Radboud University Nijmegen Medical Centre, Nijmegen, The Netherlands; Duke University Medical Center, United States of America

*Aspergillus fumigatus*, a ubiquitously distributed opportunistic pathogen, is the global leading cause of aspergillosis and causes one of the highest numbers of deaths among patients with fungal infections [1]. Invasive aspergillosis is the most severe manifestation with an overall annual incidence up to 10% in immunosuppressed patients, whereas chronic pulmonary aspergillosis affects about 3 million, primarily immunocompetent, individuals each year [2]. Three triazole antifungals, namely itraconazole, voriconazole, and posaconazole, are recommended first-line drugs in the treatment and prophylaxis of aspergillosis [3]. However, azole resistance in *A. fumigatus* isolates is increasingly reported with variable prevalence in Europe, the United States, South America, China, Japan, Iran, and India [4]–[9]. For example, about 10% of strains of *A. fumigatus* from the Netherlands are itraconazole resistant, and in the United Kingdom, the frequency increased from 0%–5% during 2002–2004 to 17%–20% in 2007–2009 [10]–[13]. In the ARTEMIS global surveillance program involving 62 medical centers, 5.8% of *A. fumigatus* strains showed elevated MICs to one or more triazoles [5]. Similarly, the prospective SCARE (Surveillance Collaboration on *Aspergillus* Resistance in Europe) study involving 22 medical centers in 19 countries identified an overall prevalence of 3.4% azole resistance. Azole-resistant *A. fumigatus* (ARAF) ranged from 0% to 26% among the 22 centres and was detected in 11 (57.9%) of the 19 participating European countries [4 and P.E. Verweij, personal communication]. Interestingly, almost half (48.9%) of the ARAF isolates from the SCARE network in European countries were resistant to multiple azoles and harbored the TR_34_/L98H mutation in the *cyp*51A gene [4 and P.E. Verweij, personal communication]. Indeed, multi-azole resistance in *A. fumigatus* due to the TR_34_/L98H mutations has become an emerging problem in both Europe and Asia and has been associated with high rates of treatment failures [12]–[14].

Azole antifungal drugs inhibit the ergosterol biosynthesis pathway, specifically the cytochrome p450 sterol 14-α-demethylase encoded by the *cyp*51A gene, which leads to depletion of ergosterol and accumulation of toxic sterols. The majority of ARAF isolates contain alterations in the target enzyme and the mutated target showed reduced or no binding to the drugs [15]. While most mutations in ARAF isolates were single nucleotide substitutions in the target gene (*cyp*51A), mutations at other genes such as the *cdr*1B have also been reported. For example, in the United Kingdom the frequency of ARAF isolates without *cyp*51A mutations has been reported to be more than 50% [16].

## Routes of Azole Resistance Development

The epidemiologic data on azole resistance is mainly from two clinical entities. One group comprises noninvasive diseases including patients with allergic bronchopulmonary aspergillosis (ABPA), aspergilloma, and chronic pulmonary aspergillosis (CPA) who were treated with long-term azole therapy (mainly itraconazole) and developed acquired resistance after 1–30 months of treatment [13]. In these patients, the ARAF isolates may be resistant to only itraconazole or exhibit a multi-azole-resistant phenotype. The underlying resistance mechanism commonly involves point mutations in the *cyp*51A gene, indicating that in patients exposed to long-term azole therapy, the fungus is capable of rapidly adapting to azole drug(s) [11]–[14]. The genotypic analysis of serial isolates of *A. fumigatus* from patients with chronic aspergillosis revealed that the initial susceptible and later resistant isolates had the same genotype. The only changes were the specific mutations conferring azole resistance, consistent with the development of resistance arising from azole therapy [13].

The second group of patients with ARAF are those with acute aspergillosis but with no known prior exposure to azole drugs [12]. In contrast to the first group in which *de novo* mutation of the fungus in cavitary lesions is the primary mechanism for the development of azole resistance, those of the second group likely acquired ARAF strains from external environments. In fact 50% of the patients with invasive aspergillosis due to ARAF are known to be azole naïve and the outcome of patients with azole-resistant invasive aspergillosis has been dismal, with a mortality rate of 88% [12]. Eighty percent of the ARAF strains from patients with invasive aspergillosis described in the SCARE network had the TR_34_/L98H mutations, which consist of a substitution of leucine to histidine at codon 98 of the *cyp*51A gene in combination with a 34-bp tandem repeat in the promoter region. These mutations enabled resistance to itraconazole and intermediate susceptibility or resistance to voriconazole, posaconazole, or both [4], [17], [18]. As described above, although the environmentally derived azole-resistant strains are predominately associated with acute invasive infections [12], [19], the same mechanism has also been reported in patients with chronic and allergic pulmonary infections [20]. For example, Denning et al. detected TR_34_/L98H and M220 mutations in 55.1% respiratory samples of CPA and ABPA patients by direct PCR and some of these patients had no prior azole therapy [20].

## Environmentally Mediated Development of Azole Resistance

Several recent findings support the hypothesis that ARAF strains in patients with invasive aspergillosis were more likely to be acquired from environmental sources rather than from *de novo* mutation and selection within patients during azole therapy. First, ARAF strains have been found in patients who had never been treated with azole antifungal drugs [7], [12]–[14]. Second, ARAF strains have been found in many environmental niches including flowerbeds, compost, leaves, plant seeds, soil samples of tea gardens, paddy fields, hospital surroundings, and aerial samples of hospitals [19], [21]–[23]. The majority of the environmental ARAF isolates harbor the TR_34_/L98H mutations at the *cyp*51A gene [19], [21]–[23]. ARAF isolates with the TR_34_/L98H mutations have been detected in the environment of the Netherlands, Denmark, India, and Iran [19], [21]–[23]. It is noteworthy that environmental surveys of ARAF from Europe reported that 12% of Dutch soil samples and 8% of Danish soil samples had the TR_34_/L98H genotype [19], [22]. Similarly, environmental surveys across India detected that 7% of all *A. fumigatus* isolates and 5% of soil/aerial samples carried the TR_34_/L98H mutation [21]. These strains showed cross-resistance to voriconazole, posaconazole, itraconazole, and to six triazole fungicides used extensively in agriculture [21].

Recently, another mutation of the *cyp*51A gene (TR_46_/Y121F/T289A) was reported in ARAF isolates from 15 patients in six hospitals in the Netherlands [24]. Interestingly, isolates with the same TR_46_/Y121F/T289A mutations were also recovered from patients' homes and backyards [24]. Apart from the Netherlands, clinical and environmental strains of *A. fumigatus* carrying the TR_46_/Y121F/T289A mutations have also been identified in neighboring Belgium [25] and in India [26]. As most patients acquire *A. fumigatus* from the environment, the emergence and spread of azole-resistant strains in the environment will put more humans at risk, especially those with compromised immunity.

## Linking Clinical Azole Resistance in *Aspergillus fumigatus* to Fungicide Usage in Agriculture

The hypothesis that clinical azole resistance in *A. fumigatus* is related to the use of fungicides in agriculture was first proposed by investigators from the Netherlands [19]. The resistant genotype TR_34_/L98H was found in 90% of ARAF isolates obtained from azole-naïve patients [11] and they hypothesized that if strains of *A. fumigatus* received sufficient azole challenge in the environment through nonmedical application of azole compounds, azole-resistant strains would be selected and spread [27]. Demethylase inhibitors (DMIs) including azole fungicides are commonly used for crop protection and for the preservation of a variety of materials such as wood [27]. For example, azole fungicides are broadly used to control mildews and rusts of grains, fruits, vegetables, and ornamentals; powdery mildew in cereals, berry fruits, vines, and tomatoes; and several other plant pathogenic fungi. Over one-third of total fungicide sales are azoles (mostly triazoles) and over 99% of the DMIs are used in agriculture. In addition, there are over 25 types of azole DMIs for agricultural uses, far more than the three licensed medical triazoles for the treatment of aspergillosis. Furthermore, the azoles could persist and remain active in many ecological niches such as agricultural soil and aquatic environments for several months.

The widespread application of triazole fungicides and their persistence in the environment are significant selective forces for the emergence and spread of ARAF. These environmental triazoles can reduce the population of azole-susceptible strains and selecting for azole-resistant genotypes [27]. Intensive use of DMI fungicides for post-harvest spoilage crop protection against phytopathogenic molds is known to cause the development of resistance in many fungi of agricultural importance. For example, resistance or tolerance to triazole fungicides has been reported for important crop pathogens such as *Mycosphaerella graminicola* (wheat), *Rhynchosporium secalis* (barley), and *Botrytis cinerea* (strawberry) [28]. Since *A. fumigatus* shares its natural environments with many fungal plant pathogens, strains of *A. fumigatus* are also exposed to the same strong and persistent pressure from fungicides. Indeed, the presence of a tandem repeat at the 5′-end upstream of the 14-α-demethylase gene is an important mechanism found in many plant pathogenic molds resistant to sterol DMI fungicides [19].

**Figure 1:**
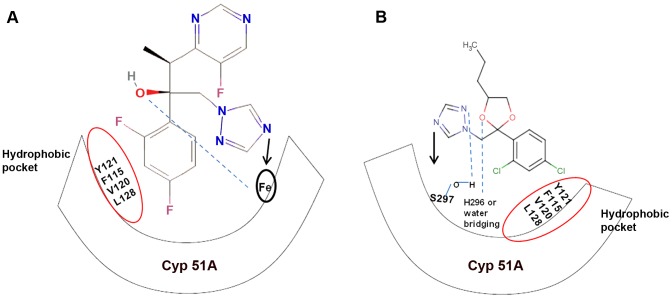
Diagrammatic representation of similar structural binding mode of medical triazoles and triazole fungicides to *cyp*51A of wild-type *A. fumigatus*. (a) Dihalogenated phenyl group of triazoles forms van der Waals contact with the hydrophobic residues (encircled in red) of the active site (*cyp*51A), and the nitrogen atom of the five-membered aromatic ring of triazoles binds to the *cyp*51A heme moiety. In addition, the D-ring propionate (C_2_H_5_COO^−^) of the heme moeity forms hydrogen bonds with the side-chain hydroxyl group of triazoles. (b) Triazole fungicides show similar van der Waals contact at the hydrophobic pocket. However, the nitrogen atom of the five-membered aromatic ring of fungicide triazoles binds to the Ser297 residue at the active site. In addition, the triazoles tebuconazole and epoxiconazole are known to interact with the His296 residue while penconazole and metconazole form water-bridging interactions at the active site.

All *A. fumigatus* isolates with the TR_34_/L98H mutations from both clinical and environmental origins have shown cross-resistance to not only all three medical triazoles, but also five agricultural triazole DMI fungicides: propiconazole, bromuconazole, tebuconazole, epoxiconazole, and difenoconazole [21], [29]. These results are consistent with the hypothesis that exposure of *A. fumigatus* to azole fungicides in the environment causes cross-resistance to medical triazoles. By molecular modeling studies, these five triazole DMI fungicides were found to have similar molecular structures as medical triazoles (Figure 1) and they all adopt a similar conformation while docking the target enzyme in susceptible strains of *A. fumigatus* [15], [29]. However, there was limited docking and activity against ARAF strains with the TR_34_/L98H mutation [29]. A similar phenomenon was also observed in the maize anthracnose fungus *Colletotrichum graminicola*. Strains of *C. graminicola* were able to efficiently adapt to media containing azoles, and those adapted to tebuconazole were less sensitive to all tested agricultural and medical azoles than the nonadapted control strain [28]. In addition, tebuconazole induced tandem repeat expansion in the promoter region of *cyp*51A in *A. fumigatus* isolates *in vitro*, indicating that fungicide pressure can rapidly select adaptive genomic changes in this mold [29]. Agriculturally selected antibiotic resistance has also been reported in many bacteria, contributing to the broad distribution of multidrug-resistant pathogenic bacteria in patients and hospitals. While horizontal gene transfer is an important mechanism for the spread of antibiotic-resistant genes among bacteria, this mechanism is not commonly found in fungal pathogens. However, similar to that in bacterial pathogens, once multidrug-resistant genotypes arise in fungal pathogens, such genotypes can spread very quickly to other geographic regions and ecological niches through vegetative cells and airborne spores such as conidia.

## Clonal Expansion and Fitness of TR_34_/L98H *Aspergillus fumigatus* Strains

A recent analysis of 255 Dutch *A. fumigatus* isolates using 20 molecular markers identified five distinct genotype groups in the Netherlands. Interestingly, all the multi-triazole resistant (MTR) isolates with the TR_34_/L98H mutation belonged to one group and, overall, they were genetically less variable than susceptible isolates [30], consistent with a single and recent origin of the resistant genotype. Similarly, all MTR *A. fumigatus* clinical and environmental strains obtained from diverse geographical regions from India belonged to a single multilocus microsatellite genotype [21]. The genotype analysis suggested that the ARAF genotype in India was likely an extremely adaptive recombinant progeny derived from a cross between azole-resistant strains migrated from outside of India and a native azole-susceptible strain from within India, followed by mutation. The abundant phylogenetic incompatibility is consistent with sexual mating in natural populations of this species in India [21].

A potential consequence of harboring the multidrug-resistant mutations in ARAF strains might be a reduced fitness in the absence of the drug as compared to the wild-type isolates. However, evidence so far suggested that the TR_34_/L98H mutation had little or no adverse fitness consequence. For example, the composite survival index (CSI) was used to measure the virulence properties of the *cyp*51A gene–associated resistance mechanism in *A. fumigatus* isolates [31]. The analyses revealed that strains with the TR_34_/L98H mutation had virulence comparable to the wild-type controls and there was no growth impairment and no reduction of virulence with the TR_34_/L98H mutation [31]. A similar finding was reported about the fitness of azole-resistant *C. albicans* strains with mutations in the *ERG*3 gene, with the mutants retaining filamentation and virulence properties [32]. The rapid dispersal of the ARAF strains with the TR_34_/L98H genotype among regions within Asia also supports the hypothesis that these strains have robust fitness in natural environments, with comparable or even higher fitness than that of wild-type strains [21], [23]. However, we would like to note that the development of azole resistance in clinical ARAF isolates carrying mutations at other loci (i.e., the non-*cyp*51A gene) could result in a reduction of virulence [33].

## Perspectives

The rapid spread of ARAF strains is jeopardizing the treatment of patients with *Aspergillus* diseases, ruling out the use of oral antifungals for these patients and leaving only the option of intravenous amphotericin B or echinocandins. Amphotericin B has significant detrimental side-effects and echinocandins are unable to completely kill or inhibit *Aspergillus* and, as such, they have only been licensed for salvage therapy of invasive aspergillosis. At present, antifungal susceptibility testing of *A. fumigatus* against azoles is not commonly performed and thus the overall threat of ARAF is not yet completely known. However, it would be beneficial to (i) have an active multi-azole susceptibility testing of *A. fumigatus* to monitor the extent of the problem, (ii) reduce agricultural use of triazole DMI fungicides, and (iii) use combination drug therapy when dealing with infections by *A. fumigatus* strains to limit the emergence of resistance. Indeed, the European Center for Disease Prevention and Control (http://www.ecdc.europa.eu/en/publications/Publications/Forms/ECDC_DispForm.aspx?ID=1064) recommends increased surveillance for clinical and environmental azole-resistant pathogens and to conduct field trials to study the impact of nonmedical azole use in the development of azole resistance in patients. A more judicious use of azoles in patients, in agriculture settings, and alternative strategies such as chemosensitization and/or a shift from the use of purely chemical methods to a more integrated crop management approach could help lower the dosage levels of fungicides in the environment and minimize the emergence and spread of ARAF.

**Figure 2:**
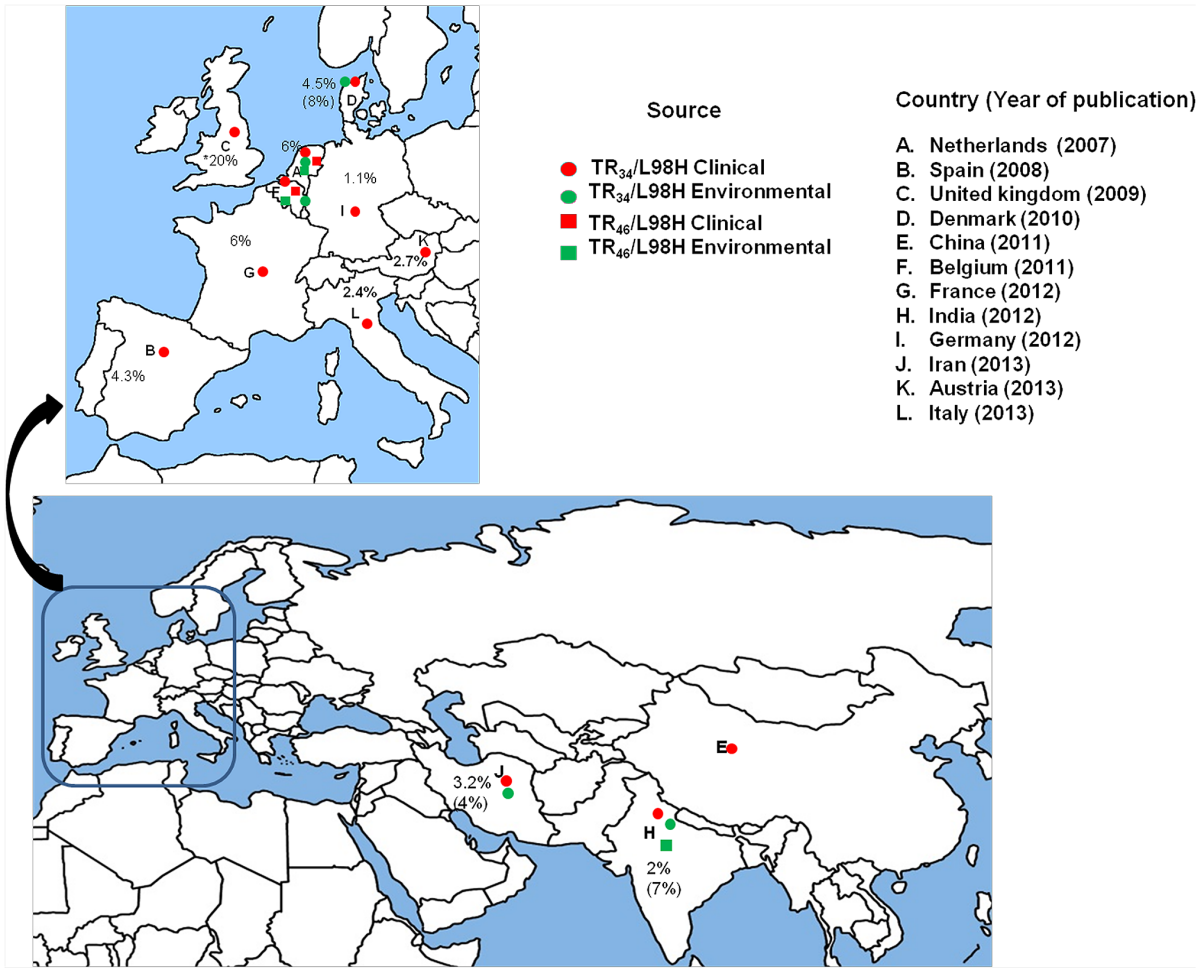
A global map depicting geographic distribution of multi-triazole-resistant clinical (red) and environmental (green) *Aspergillus fumigatus* strains carrying the TR_34_/L98H (circle) and the TR_46_/Y121F/T289A mutations (square). Countrywide prevalence rates (%) of *A. fumigatus* carrying TR_34_/L98H are presented excepting the United Kingdom, where overall azole resistance is illustrated. The percent in parentheses denotes environmental prevalence rates.

Although there are substantial data suggesting that agricultural use of fungicides have driven the emergence and spread of multi-triazole-resistant strains of *A. fumigatus*, conclusive evidence linking agricultural triazole fungicides to the emergence of TR_34_/L98H or TR_46_/Y121F/T289A genotypes in controlled field experiments is lacking. The definite evidence will help the regulatory authorities with formulation of policies to control the environmental-driven azole resistance. The two major types of azole-resistant mutations were first detected in 1998 and 2009 respectively and both are now spreading quickly (Figure 2). It is highly likely that other types of mutations conferring multiple azole resistance could emerge in the near future from environmental sources and spread among human populations.
