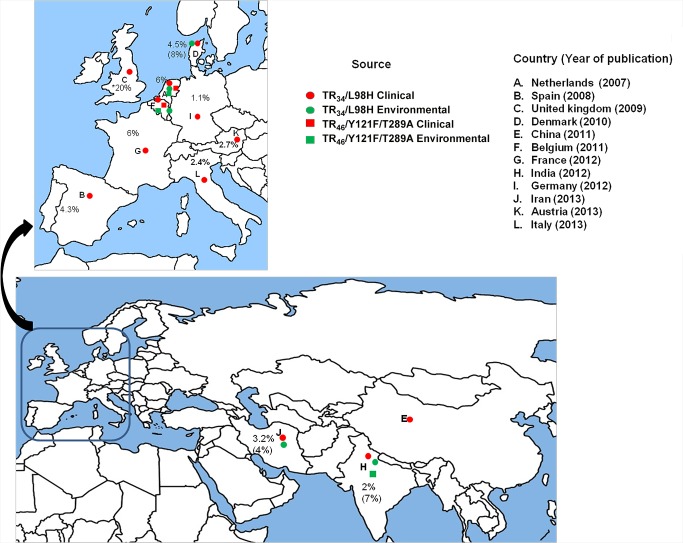# Correction: Emergence of Azole-Resistant *Aspergillus fumigatus* Strains due to Agricultural Azole Use Creates an Increasing Threat to Human Health

**DOI:** 10.1371/annotation/4ffcf1da-b180-4149-834c-9c723c5dbf9b

**Published:** 2013-11-14

**Authors:** Anuradha Chowdhary, Shallu Kathuria, Jianping Xu, Jacques F. Meis

There is an error in the labeling in Figure 2. The figure legend is correct. 

Please see the corrected figure at the following link:

**Figure:**